# The COVID-19 e-lective: using innovation to manage disrupted medical student clinical placements

**DOI:** 10.1186/s12909-023-04067-w

**Published:** 2023-02-06

**Authors:** Janie Dade Smith, Peter D. Jones

**Affiliations:** Faculty of Health Sciences and Medicine, Bond University, Gold Coast, Queensland 4229 Australia

**Keywords:** COVID-19 e-lective, Virtual tutorials, Innovative learning, Evaluation, COVID project, Disrupted clinical placements

## Abstract

**Background:**

The COVID-19 pandemic changed the way we work, spend, live, and learn. The impact was felt in the health sector where hospitals cancelled elective surgery, put on hold outpatient services, and implemented new social distancing procedures and telehealth systems, to enable hospitals to increase bed capacity. For medical students, these factors meant significant disruption to their clinical placements, remote delivery of their education, cessation of international and interstate placements, complicated by significant travel restrictions and border closures. There were concerns that final year students might be unable to graduate that year due to this lack of clinical exposure.

**Innovation:**

As a result of this disruption in late March 2020 we developed an innovative 6 week ‘COVID-19 e-lective’ rotation, consisting of online modules, virtual clinical tutorials and a COVID project totalling the equivalent of 200 h of work.

**Results:**

An evaluation was undertaken that found it to be remarkably successful in meeting the students’ learning needs and alleviating concerns about disrupted placements. It was also conducted during 2021 for all Year 4 students to help expand clinical placement opportunities.

**Outcomes:**

This paper describes the e-lective, its innovations, its challenges, and its evaluation findings, for others to learn from.

**Supplementary Information:**

The online version contains supplementary material available at 10.1186/s12909-023-04067-w.

## Introduction

The world as we know it changed in early 2020 with the pandemic of the COVID-19 virus. This virus changed the way we view the world and how we work, spend, learn, and play within it. The rules of engagement changed daily affecting everyone’s movements with the closure of international and state borders, resulting in people working remotely at home, and the closure of business and industry creating a serious economic impact.

The impact was felt none more so than in the education and health sectors, where hospitals cancelled elective surgery, put on hold outpatient services and implemented new social distancing procedures and telehealth systems, to enable hospitals to increase bed capacity for an expected wave of COVID-19 cases [1]. For medical students this meant a significant disruption to their clinical placements, remote delivery of their education, cessation of international and interstate placements due to significant travel restrictions and border closures [2]. This was universal globally with leading medical organisations providing principles [3] and guidance statements [4] for medical student involvement and employment during the COVID-19 pandemic, with the United Kingdom expediating some graduations to boost the workforce [5]. The Medical Deans of Australia and New Zealand recommended that no medical students should work in high risk and high stress areas within health services, and that clinical placements in these areas should cease [6]. This guidance varied between countries and Australian states, with New South Wales (NSW) allowing students to work in employed roles, and other states not, due to safety concerns and access to Personal Protective Equipment (PPE).

Bond University on the Gold Coast in Australia sits on the border of NSW and Queensland and many medical student clinical placements closed in public and private hospitals, general practices as well as all international and cross border placements to ensure student safety. The final two clinical years of the Bond medical program consists of 6 × 7-week clinical rotations annually and there were serious concerns that over half of Bond’s final year 5 cohort (*n* = 103) may not have sufficient clinical experience to graduate in 2020; as well as significant disruption to Year 4 students during their first clinical year (*n* = 123), their selective and speciality rotations in private hospitals.

While these disruptive factors were concerning it was also a time for real innovation, which many other universities were also doing [7]. This paper reports on what we did, which others could replicate.

Ethics was obtained to complete the study called “The COVID-19 e-lective: using innovation to manage disrupted medical student placements.” Approval to proceed with the study was granted by the Bond University Human Research Committee. The ethics approval number was HREC/PJ00027/2022. We confirm that all data obtained for this study was obtained in accordance with the relevant guidelines and regulations. The ethics approval allows for the inclusion of de-identified student evaluations in this paper. Informed consent was obtained from all subjects and/or their legal guardians involved in the project.

## The COVID-19 e-lective - what did we do?

As a result of this disruption in late March 2020 we developed the ‘COVID-19 e-lective’, which commenced on 28th April 2020 for those affected students (*n* = 56). The goals of the COVID-19 e-lective were to provide a feasible, robust, rigorous, investigative, innovation and contemporary elective that enabled the students to make the current clinical world relevant, cover key content to assure intern preparedness, and to explore how COVID-19 had changed one key area of medical practice. To ensure academic equivalence to a clinical rotation, students engaged in approximately 200 h of activity over a 6-week period, with one extra week for preparation.

An initial brainstorming session helped identify what an e-lective could cover to meet learning goals. It was important to identify a variety of interactive tasks, as well as the mechanisms that could make it feasible, and enjoyable, and take advantage of the shift to online education. An overarching aim was to provide structure and support so that students could be fully engaged across the full 6 weeks of curriculum time that was occupied by the COVID e-lective.

Four principal areas to meet the goals of the e-lective were identified:

1. The National Prescribing Service Modules (35 hours) – over twenty online modules on commonly encountered topics such as atrial fibrillation, depression in adolescents and hypertension.
2. Clinical knowledge and online materials (35 hours) – a list of COVID-19 and other modules for students to choose from to meet their learning gaps and areas of interest – OSLER app [8]: prone ventilation, using PPE, COVID podcasts; or Online Med-Ed (http://onlinemeded.org/) with over 250 topics across 19 specialities, Cochrane guidelines, Melbourne University COVID-19 Lung Ultrasound course, and WHO COVID-19 modules [9] and many others as they emerged.
3. Clinical communication and procedural skills (30 hours) – weekly virtual tutorials using cases studies, podcasts, videos, PowerPoint Presentations facilitated virtually by clinical tutors using the Microsoft Teams platform [10].
4. COVID-19 e-lective project (100 h) – from eight topic areas with triggers.

The COVID e-lective model was also flexible enabling those students who could undertake part of their clinical placement to only enrol in part of the COVID e-lective and excluding the project component.

A search of the internet was undertaken to identify potential relevant COVID online modules and resources, which were found to be abundant, mostly free and being produced daily.

A ‘COVID e-lective Student Guide’ [11] was then developed that outlined the goals of the e-lective, the learning outcomes, core components, assessment requirements, roles and supporting materials. Contact was made with potential and available clinical tutors (*n* = 7) and project supervisors (*n* = 15), many of whom volunteered their time. Two academics developed and coordinated the COVID-19 e-lective program in consultation with a small team of academics and professional support officers communicating from home via Microsoft Teams platform (Microsoft Teams, 2020). Assessment processes were established using the OSLER [8] platform, requiring students to submit achievement of the various components and using electronic marking.

## Innovations developed as part of the COVID e-lective

### The “Clinical” Tutorials:

An innovative component of the program were the virtual clinical tutorials and clinical skills development sessions, equivalent to 5 hours of student learning weekly. The aim was to provide a trigger for students to perform clinical tasks to help develop the clinical communication skills required to work as an intern. We developed weekly podcasts, to guide tutors and students, case studies, and voice over PowerPoint presentations. The sessions were designed as ‘flipped classrooms’ where students were expected to be fully prepared prior to each weekly virtual tutorial. The 5 hours weekly consisted of the time taken by the student to review the stimulus materials, complete the required pre-tutorial tasks, participate in the tutorial and to revise their learning outcomes through listening to the podcast or conducting any activities set by their tutor. Attendance was compulsory and was assessed by the clinical tutors as a pass/fail hurdle assessment. The virtual tutorials provided personal ‘face to face’ student tutor interaction.

Written and verbal informed consent was obtained from the patient and/or their guardian prior to the recording of each of the clinical interactions used in the tutorial series. An example of a case used for the Clinical Tutorial and the tasks students would be required to complete and discuss is shown in Case Example 1. No written identifying information was recorded for each patient interaction. Ethics for the evaluation of the Covid Elective was approved by the institution’s ethics committee approval number HREC/PJ00027/2022.

### The COVID project:

The second innovative component was the COVID project (100 h). It provided students with eight topic options – global health, public health, child health, aged care, legal and ethical issues, general practice and primary health care, mental health, and evidence-based practice. Each topic area was allocated one or two project supervisors who met weekly with 6–8 students on Microsoft Teams to discuss process, progress and flesh out ideas. A list of triggers and questions were developed in the Student Guide to stimulate the students thinking and author guidelines were provided. An example such as ‘how can you isolate at home when you do not have a home?” – resulted in an excellent paper on homelessness in Melbourne in the COVID era compared with the UK approach. Many students undertook cases studies using BMJ guidelines, or comparative studies of various countries to examine the different COVID approaches. Other project examples included: a case study of the Singapore experience of migrant workers and the incidence of COVID-19 [12]; obstetric care comparing the COVID obstetric guidelines in three countries and resultant health outcomes; a group systematic review of the impact of COVID-19 on mental health; and a clinical audit exploring the reduction in paediatric admissions during the pandemic. Several students achieved publication of their project work [12–16].

A great deal of goodwill was provided by the virtual clinical tutors (*n* = 7) and the project supervisors (*n* = 15) who were rounded up voluntarily from academic staff and clinicians at the beginning of the project. In this paper we describe the innovation and how it was received by the students who undertook this virtual placement.

## Research methods

An evaluation was undertaken at the end of each rotation between 2020 and 21. The evaluation was approved by the Bond University Human Research Committee approval number HREC/PJ00027/2022.

At the end of each rotation students were invited to participate in the evaluation of the rotation. Participation in the evaluation was anonymous and voluntary.

The evaluation consisted of two parts.

**Fig. 1:**
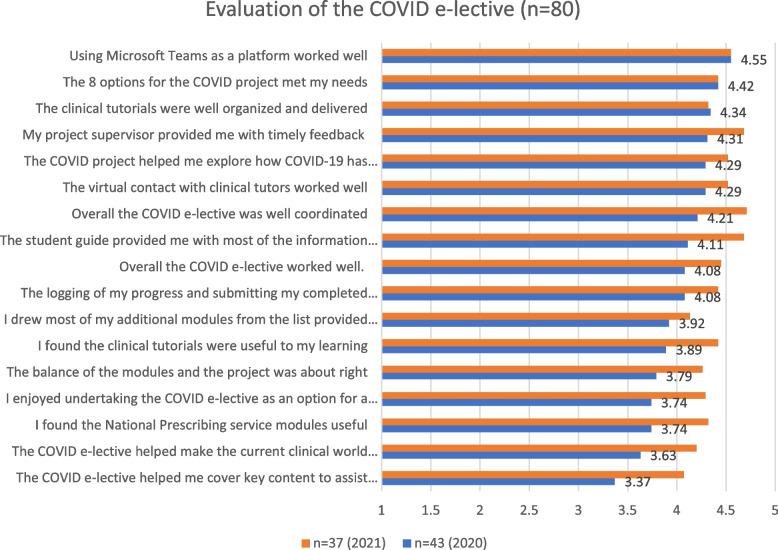
Evaluation of the COVID-19 e-lective

The first part was quantitative and included an 18-question survey where students were able to answer using a 5-point Likert scale with responses of ‘5-Strongly Agree’ and ‘4-Agree’, ‘3-Nuetral’, ‘2-Disagree’, and ‘1-Strongly Disagree’: The results of the quantitative evaluation are presented in Fig. 1 at the end of the paper.

The second part of the evaluation was qualitative. The students were given the opportunity to answer two open ended questions about the strengths and suggested improvements of the rotation using the Survey monkey platform. In the first question students were asked to list their two favourite activities completed during the rotation. The second questions asked students to make two suggestions to help improve the COVID-elective. The results of the two open-ended questions were common themed for analysis.

## Evaluation results

250 students completed the COVID e-lective between April 2020 and November 2021. The course was run 10 times between April 2020 and November 2021 in parallel with the clinical placement schedule of Medical Program of Bond University.

The COVID elective was compulsory for all year four students in 2020 and 2021.

Final year (Year 5) students were prioritised for any available clinical elective placements in 2020 to ensure they were ready to commence Internship on graduation. Only a small number of Year five students completed the COVID elective. No year 5 students completed the COVID e-lective in 2021.

Of the eighty students who completed the evaluation of the whole COVID-19 e-lective, seventy-seven were fourth year students and three were final year students. The overall response rate was 32% (80 of 250).

Overall, the students felt the COVID e-lective worked very well (M = 4.27), it was well coordinated (M = 4.46), it provided a good option for a disrupted placement (M = 3.99), the 8 project options met their needs very well (M = 4.42), allowed them to explore issues of interest to them (M = 4.44) and the projects were very well supervised with timely feedback (M = 4.5). Students felt the clinical tutorials we useful to their learning (M = 4.15), were very well organised (M = 4.33), the Teams Microsoft platform worked very well (M = 4.55) and the logging of progress for assessment using OSLER worked well (M-4.29). They felt the NPS, and the list modules were good (M = 4.03), and many drew other modules that they identified as they emerged. The students felt the COVID e-lective as OK (M = 3.72) in meeting key content, it made the current clinical world relevant (M = 3.91) and assisted in their intern preparedness (M = 3.72). Overall, these are incredibly positive outcomes, and there was a significant improvement noted between the 2020 and 2021 results see Fig. 1. This is due to a review of the project options during implementation and changes made because of the findings from the 2020 evaluation results.

Students were also asked to list the two best things about the e-lective; and forty-one responses were received. The three best things identified were the virtual clinical tutorials, the projects, and opportunities to work at home in self-directed learning activities (*n* = 22 respectively).

*I very much enjoyed the project as it allowed me to develop a deeper understanding about COVID in aspects that I would not have previously learnt about and look at more current research. It was also a good practise for research and writing professional articles. I also thoroughly enjoyed the sessions with the tutor as the discussions were very interesting and it was good to have the tutor’s clinical perspective on current topics of healthcare.*

*Opportunity to research an element of COVID-19 that interested me, and small groups lead by a tutor.*

*I really enjoyed the tutorials as it was a space where various questions could be answered, and intern-specific queries could be discussed.*

Students also enjoyed learning about COVID-19 (*n* = 8), which was viewed as relevant to current times, catching up on work in their area of interest, providing them with flexibility (*n* = 6), and the quality of the project supervisors (*n* = 8).

*The ability to tailor the COVID project to something of my interest. My project supervisor (name) gave timely feedback. The clinical tutorials were helpful to my clinical knowledge and run well* via *TEAMs and in small groups. My tutor (name) was fantastic!*

*Opportunity to engage in a project, learning about COVID, patio physiology and treatment options was really fascinating. (Supervisor name) gave fantastic explanations of all these concepts.*

Students identified areas of improvement and thirty-eight responses were received. They would have liked more guidance on the modules (*n* = 11), more COVID related information in the tutorials (*n* = 5), more academic writing for publication assistance (*n* = 4) and some found the OSLER and NPS modules a bit dry.

## Challenges

There were several challenges. We moved from conception to implementation of this COVID-19 e-lective rotation in under 3 weeks; while we all worked from home and introduced remote learning. We also needed to identify and develop a range of clinically relevant and useful activities i.e., podcasts, case studies, videos, PowerPoint presentations, online courses, and modules, as well as finding suitable project supervisors (*n* = 8 to 15 each rotation) and clinical tutors (*n* = 7).

This required engagement and goodwill from both academics and clinicians who each volunteered approximately 10 hours of their time per rotation; as well as administrative challenges to identify disrupted placements and students. Allowing several project options also meant that we could distribute the students across many potential project supervisors, each with an interest in COVID-19. This equated to approximately 1000 hours of time, in addition to the input of the two academic program co-ordinators, being provided by clinician and academic partners pro bono to deliver the COVID e-lective across 2021. In the second half of 2021 there was understandable fatigue observed amongst those colleagues who had so willing donated their time to assist the medical program through the peak of the crisis that COVID-19 presented to the delivery of undergraduate medical education. It was clear that if the medical program continued with the COVID e-lective beyond 2021 further investment in dedicated human resources would have been required.

This was also the first time a ‘clinical’ placement was run remotely using virtual platforms for delivery and assessment, and we rapidly designed and established the capacity to monitor and assess using the virtual OSLER platform. Bond University was not alone in trying to develop an alternative educational experience to the traditional face to face immersion in the clinical workplace that is the cornerstone of the final 2 years in medical school in Australia, the USA and around the world. In the Philippines, [17], medical students adapted to the impact of the pandemic in creative ways to simulate the clinical environment.

This rotation was developed with enthusiasm and adrenaline in the heat of an emerging crisis. It was able to be sustained through the enthusiasm and support of both the students who completed the placement and the academics and clinicians who ensured its effective delivery. By the end of 2021, although the impact and disruption of the COVID-19 was not fully over in Australia, it was clear that the Medical Program needed to focus on increasing face to face work-place based immersion for senior medical students and that there would no longer be a need for a “virtual” clinical placement in 2022.

So, after almost 2 years the COVID e-lective ceased and was replaced by a new community placement in January 2022.

## Conclusion

The COVID-19 e-lective proved to be a successful way to meeting the student learning needs and alleviate the concerns and provide a learning opportunity for 250 students whose clinical placement was disrupted by the pandemic. The COVID e-lective was an essential part of several strategies that were implemented by the Medical School to ensure the clinical program was able to continue throughout the Pandemic.

Although the COVID e-lective was not delivered in 2022 the course outline was retained by the Medical was retained. The medical program made the decision that having an option for students who encounter difficulty for whatever reason during their clinical rotations that allows them to remotely continue their studies without deferring for a period was a particularly useful innovation for running a medical school.

There are a variety of personal and health reasons why a student may not be able to attend a clinical placement face to face for a short period during the final 2 year of medical school but might still be able to complete an approved on-line substitute. Retaining this flexibility is a lasting and positive legacy of our school delivering this program during the height of COVID pandemic. The authors strongly recommend that all schools look at the innovations they implemented during COVID and assess which might be useful to hold on to into the future.

The key to success of this learning option was the variety that students were able to experience when completing the COVID elective. This was only possible because the authors achieved engaged with a wide number of Faculty who each provided a small amount of input into the subject over a prolonged 20-month period. The surprisingly positive evaluation is an endorsement for teamwork and sharing workload sustainably amongst staff.

## Case example 1

A discharge consultation on a 17 month old child who had suspected meningitis

A. Video Trigger

- A 12 minute video of an actual hospital consultation between a paediatrician and a mother with a 17 month old child and his mother was uploaded to the teaching platform that students could view and then prepare for an interactive tutorial

B. KeyPatient History facts that emerge from the Consultation:

1. Connor presented to hospital with a 24-hour history of lethargy and high fever
2. He was assessed as clinically having meningitis
3. The Initial work up showed he had a CSF with 596 white cells that were 58% PMN and had an CRP of 232
4. The patient remained unwell and probably photophobic 4–5 days
5. On day 3 a blood culture became positive for Staph Hominis (A coagulase negative staphylococcus)
6. The decision was made to treat empirically for suspected Bacterial Meningitis for 10 days until April 18
7. On Saturday 18th April he was assessed on the ward prior to his discharge. This discharge consultation is what the students were able to view

III. Clinical Tasks that students would complete before the Example 1 tutorial:

1. Please write the notes for today’s ward round
2. Please write a discharge summary for the GP
3. Connor is to have a hearing test in 4 weeks time. How does meningitis cause hearing loss?
4. Describe the developmental milestones demonstrated in the consultation
5. What other information may have been helpful in trying to determine whether this was bacterial meningitis or not?
6. Do you think the positive blood culture for staph hominis is important in this case? Look up the literature and argue your answer on the basis of available medical evidence.

IV. Format of the Clinical Tutorial

1. 6–8 students were allocated a medical practitioner who would be the tutor discuss the clinical case with them using Mirosoft Teams©. For each clinical rotation there were three clinical tutorial tutors who taught and assessed the students.
2. At the beginning of each week the clinical cases and expected tasks were made available on the learning platform to the students.
3. On the morning of the tutorial students were expected to email the tutor the work they had completed on the case and their answers to each of the questions.
4. The clinicain would ensure each student would be able to present and be the key person expected to lead discussion about each of the questions/activties. At the end of the tutorial students would be given learning issues they would be expected to research before the next tutorial.
5. Students were assessed as pass/fail on the basis of them participating in all steps of the tutorial.

## Supplementary Information

**Additional file 1.**

## Data Availability

All Data are stored as deidentified by Bond University for 7 years from the time of collection. Data from the survey monkey has been uploaded and is available for assessment. Further data will be made available on request by Professor Janie Dade Smith through contacting her on jansmith@bond.edu.au.
