# Supplementary material for: The COVID-19 e-lective: using innovation to manage disrupted medical student clinical placements
Source: BMC Med Educ. 2023 Feb 6;23:92. doi: 10.1186/s12909-023-04067-w (PMC9901062; doi:10.1186/s12909-023-04067-w)
Supplement: Supplementary file 1 — Additional file 1. [file 12909_2023_4067_MOESM1_ESM.pdf]

Q1 What year group are you in

Answered: 37 Skipped: 0

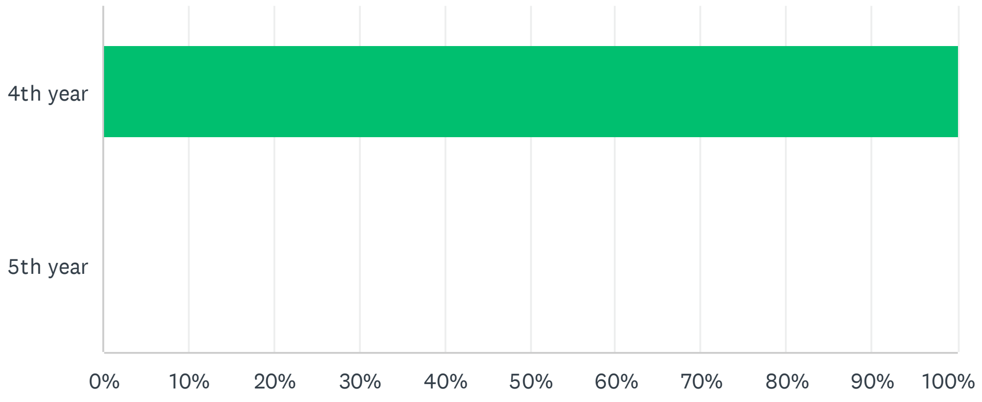

ANSWER CHOICES	RESPONSES	
4th year	100.00%	37
5th year	0.00%	0
TOTAL		37

Q2 The COVID e-lective helped make the current clinical world relevant.

Answered: 30   Skipped: 7

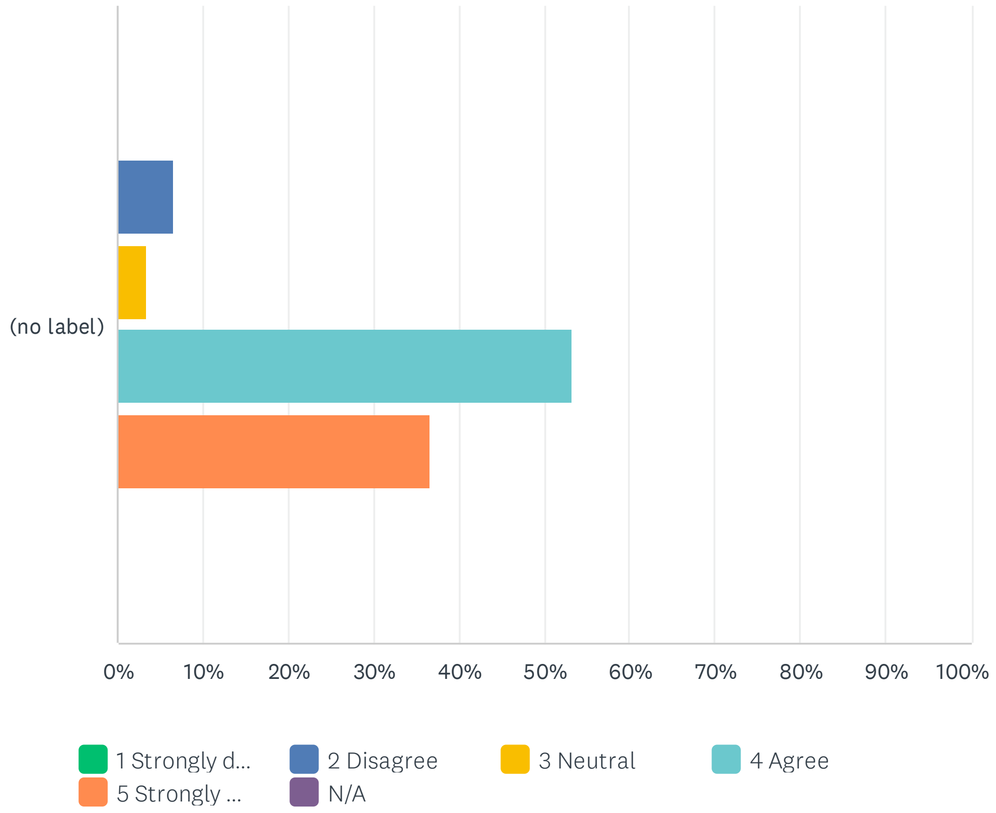

	1 STRONGLY DISAGREE	2 DISAGREE	3 NEUTRAL	4 AGREE	5 STRONGLY AGREE	N/A	TOTAL	WEIGHTED AVERAGE
(no label)	0.00%<br>0	6.67%<br>2	3.33%<br>1	53.33%<br>16	36.67%<br>11	0.00%<br>0	30	4.20

Q3 The COVID e-lective helped me cover key content to assist with my intern preparedness

Answered: 30 Skipped: 7

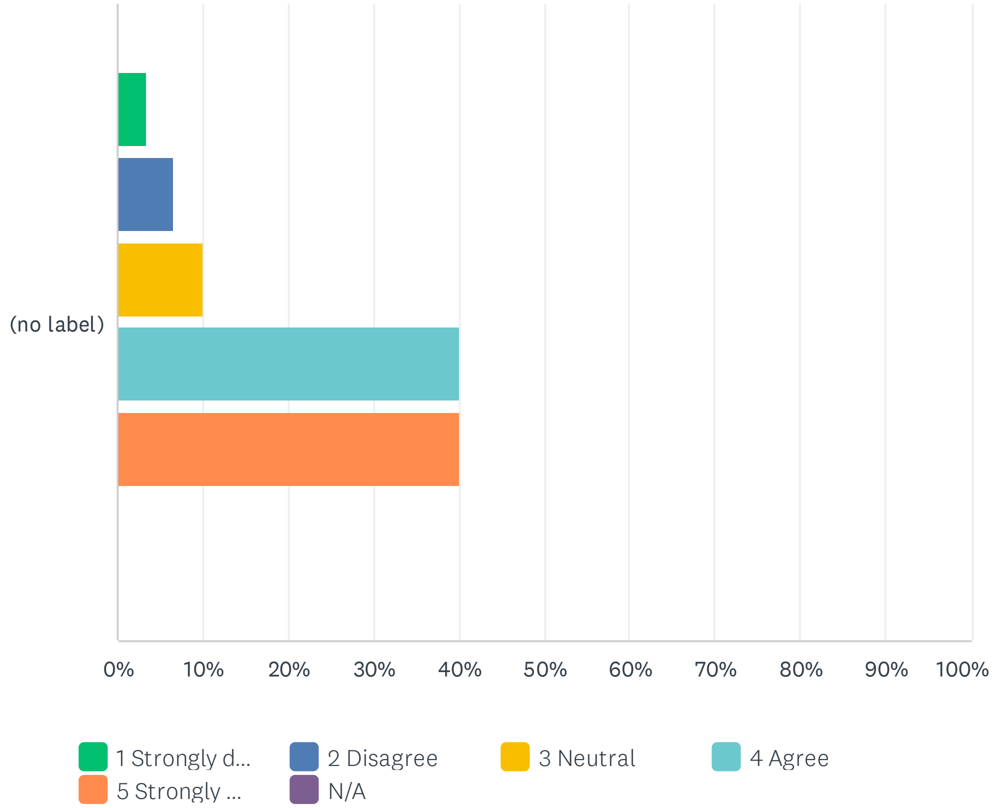

	1 STRONGLY DISAGREE	2 DISAGREE	3 NEUTRAL	4 AGREE	5 STRONGLY AGREE	N/A	TOTAL	WEIGHTED AVERAGE
(no label)	3.33%<br>1	6.67%<br>2	10.00%<br>3	40.00%<br>12	40.00%<br>12	0.00%<br>0	30	4.07

Q4 The balance between the modules, the clinical tutorials and the project was about right

Answered: 31 Skipped: 6

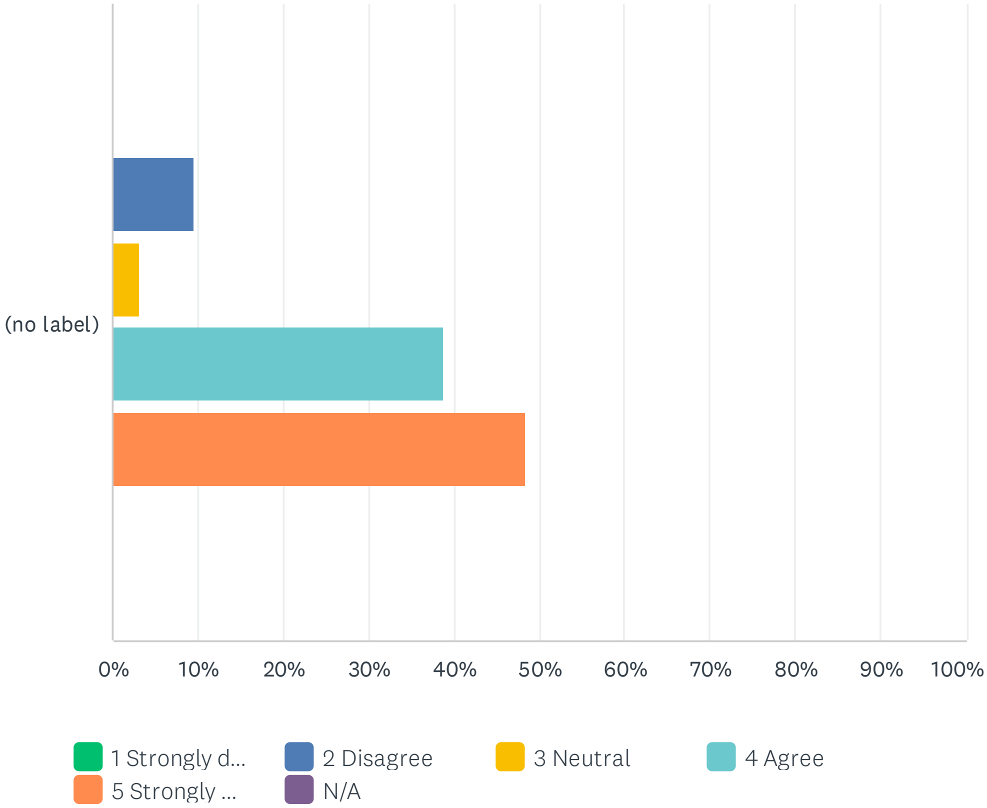

	1 STRONGLY DISAGREE	2 DISAGREE	3 NEUTRAL	4 AGREE	5 STRONGLY AGREE	N/A	TOTAL	WEIGHTED AVERAGE
(no label)	0.00%<br>0	9.68%<br>3	3.23%<br>1	38.71%<br>12	48.39%<br>15	0.00%<br>0	31	4.26

Q5 The student guide provided me with most of the information I needed

Answered: 31 Skipped: 6

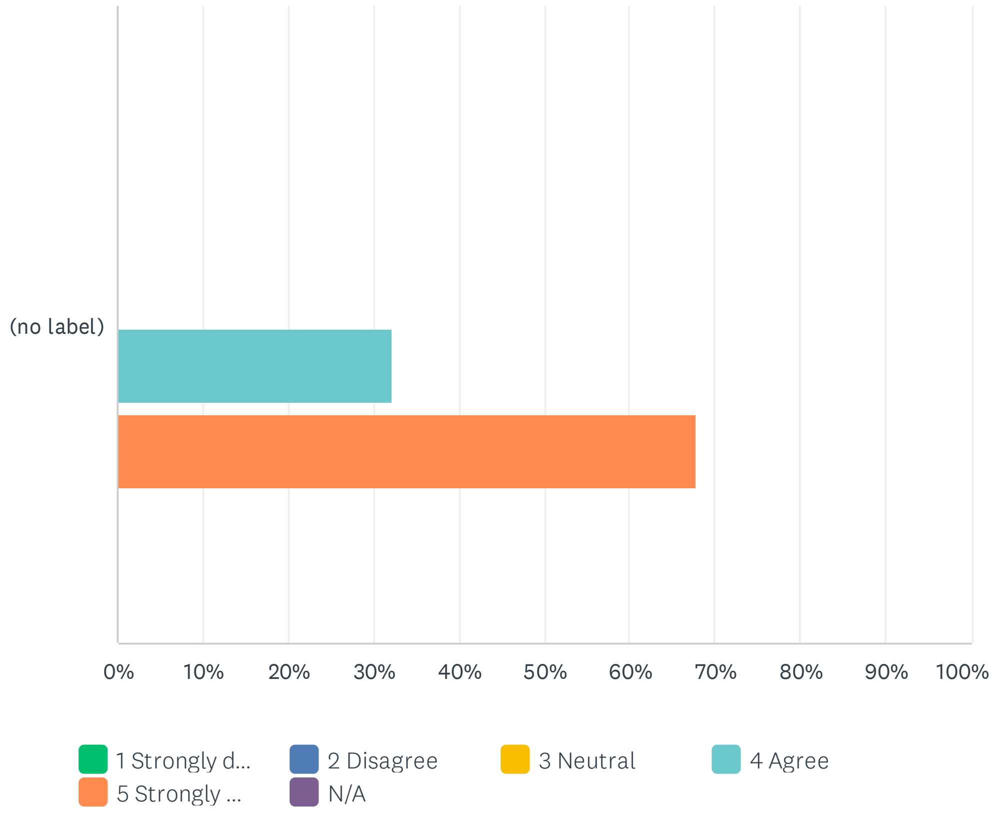

	1 STRONGLY DISAGREE	2 DISAGREE	3 NEUTRAL	4 AGREE	5 STRONGLY AGREE	N/A	TOTAL	WEIGHTED AVERAGE
(no label)	0.00%<br>0	0.00%<br>0	0.00%<br>0	32.26%<br>10	67.74%<br>21	0.00%<br>0	31	4.68

Q6 I found the National Prescribing Service and the OSLER modules useful

Answered: 31 Skipped: 6

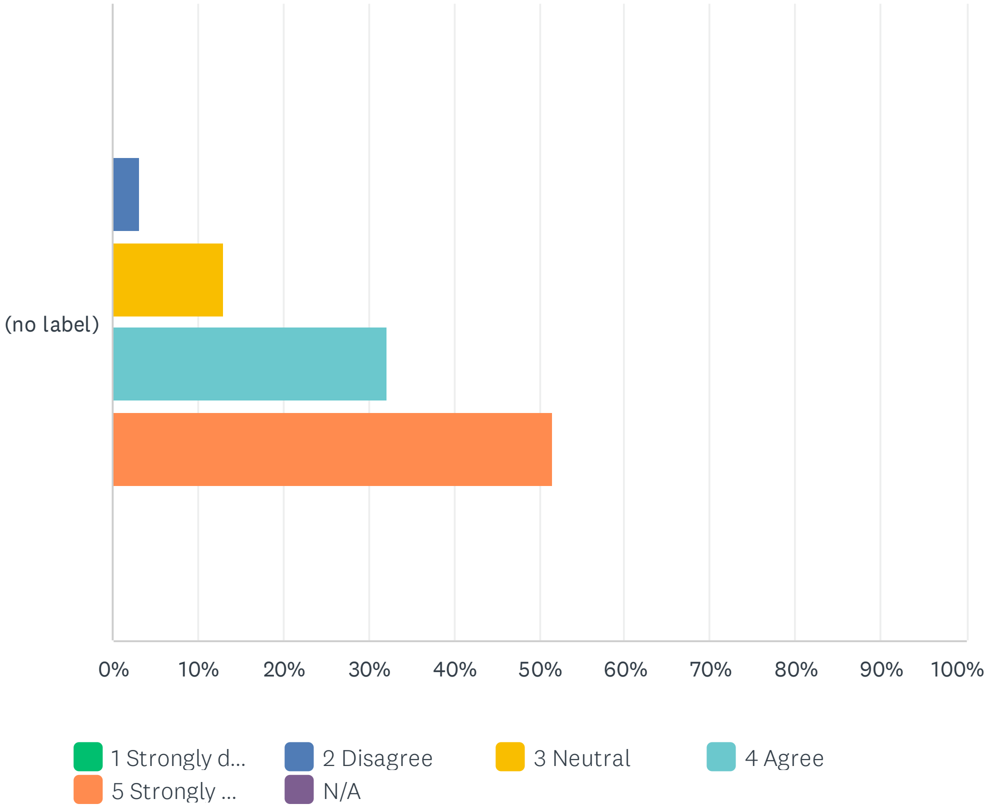

	1 STRONGLY DISAGREE	2 DISAGREE	3 NEUTRAL	4 AGREE	5 STRONGLY AGREE	N/A	TOTAL	WEIGHTED AVERAGE
(no label)	0.00%<br>0	3.23%<br>1	12.90%<br>4	32.26%<br>10	51.61%<br>16	0.00%<br>0	31	4.32

Q7 I drew most of my additional modules and courses from the list provided in the student guide

Answered: 31 Skipped: 6

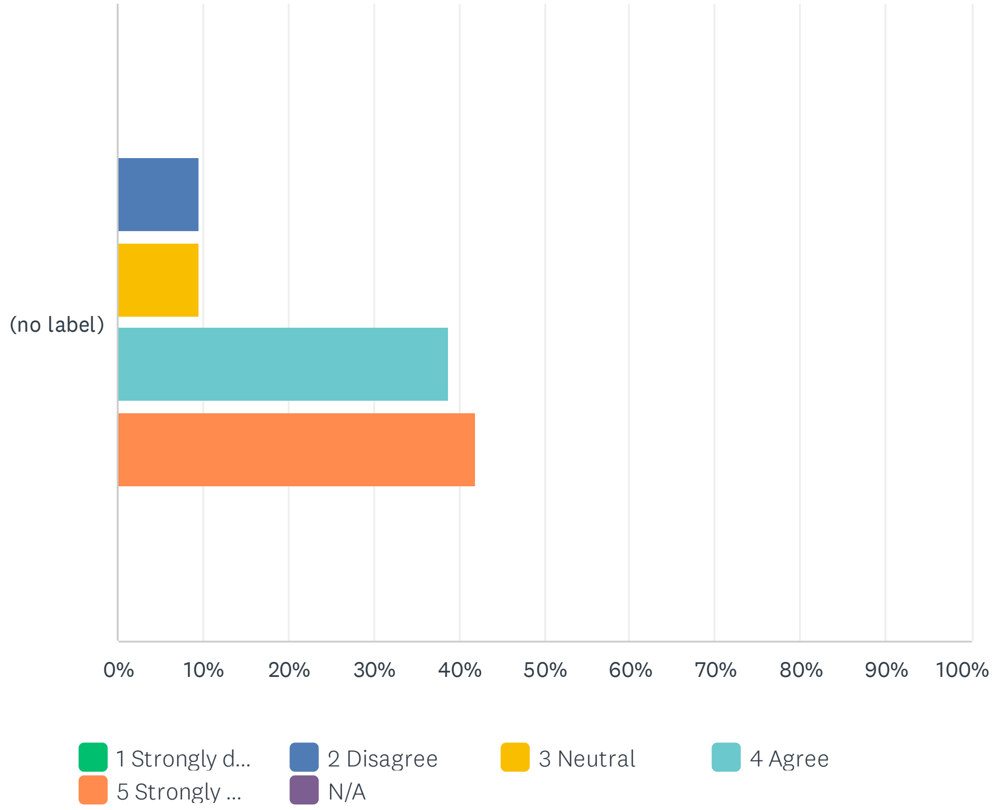

	1 STRONGLY DISAGREE	2 DISAGREE	3 NEUTRAL	4 AGREE	5 STRONGLY AGREE	N/A	TOTAL	WEIGHTED AVERAGE
(no label)	0.00%<br>0	9.68%<br>3	9.68%<br>3	38.71%<br>12	41.94%<br>13	0.00%<br>0	31	4.13

Q8 I found the clinical tutorials were useful to my learning

Answered: 31   Skipped: 6

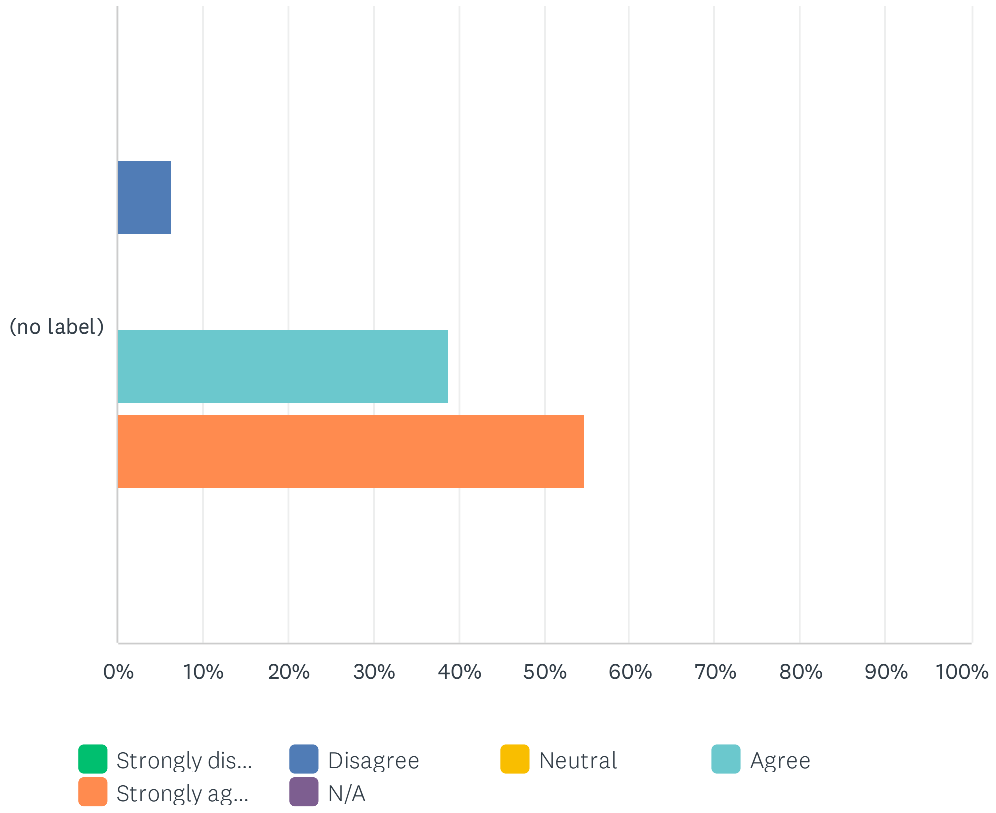

	STRONGLY DISAGREE	DISAGREE	NEUTRAL	AGREE	STRONGLY AGREE	N/A	TOTAL	WEIGHTED AVERAGE
(no label)	0.00%<br>0	6.45%<br>2	0.00%<br>0	38.71%<br>12	54.84%<br>17	0.00%<br>0	31	4.42

Q9 The clinical tutorials were well organized and delivered

Answered: 31 Skipped: 6

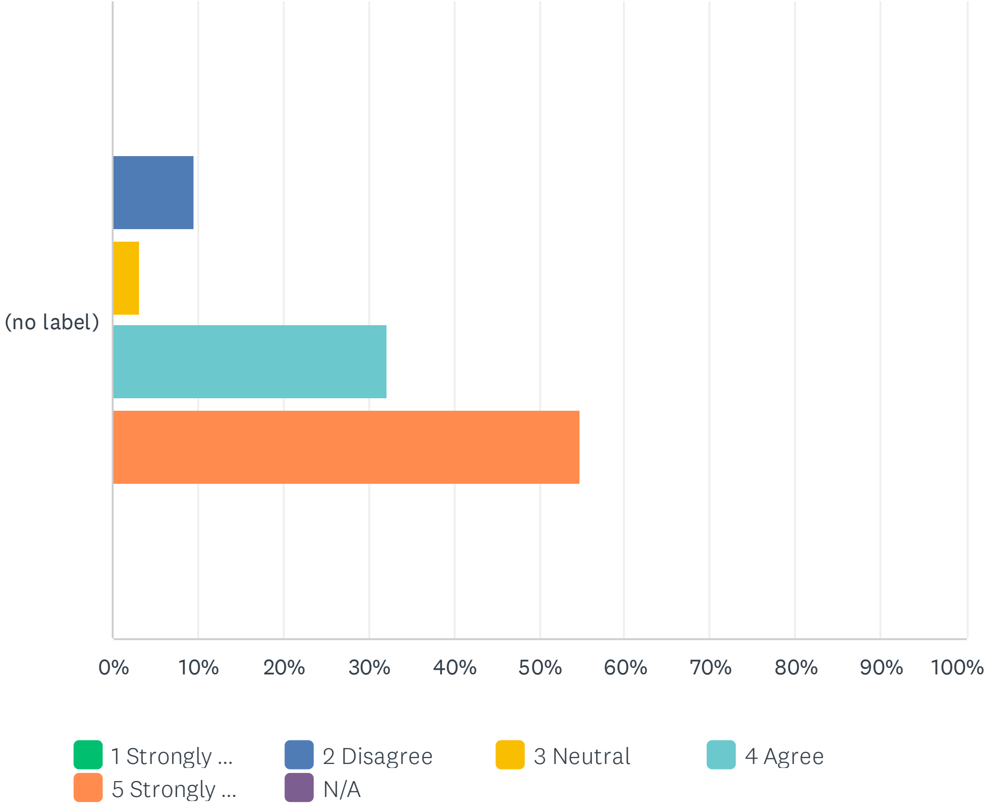

	1 STRONGLY DISAGREE	2 DISAGREE	3 NEUTRAL	4 AGREE	5 STRONGLY AGREE	N/A	TOTAL	WEIGHTED AVERAGE
(no label)	0.00%<br>0	9.68%<br>3	3.23%<br>1	32.26%<br>10	54.84%<br>17	0.00%<br>0	31	4.32

Q10 The virtual contact with clinical tutors worked well

Answered: 31 Skipped: 6

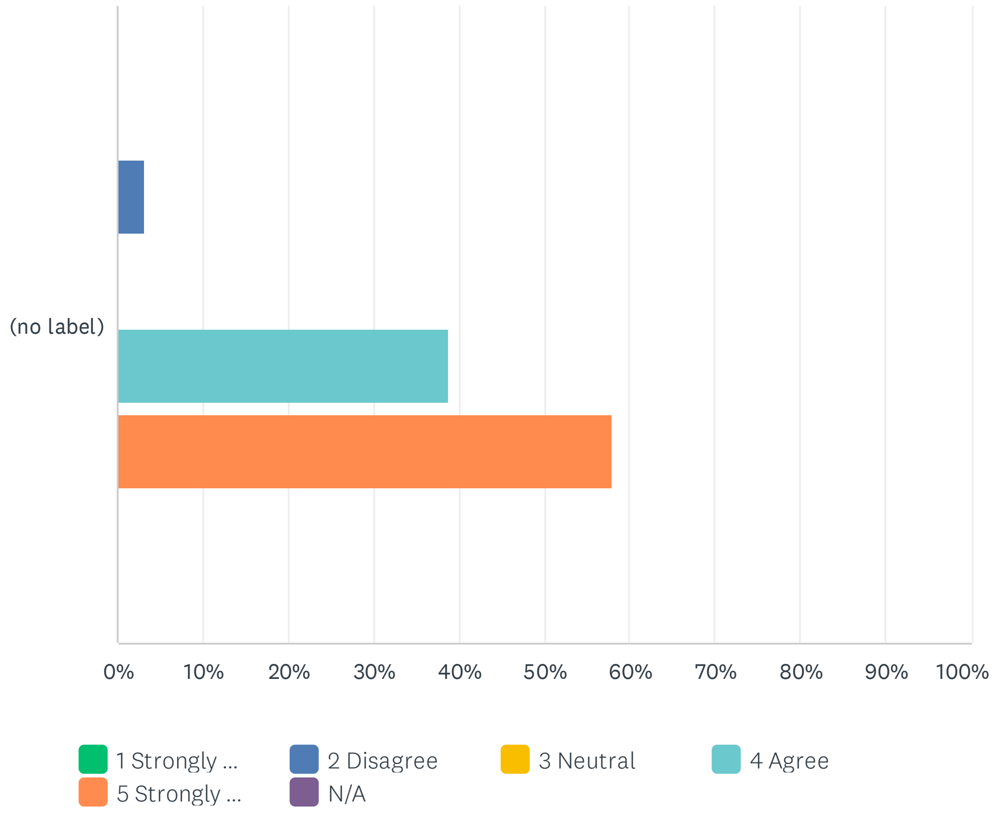

	1 STRONGLY DISAGREE	2 DISAGREE	3 NEUTRAL	4 AGREE	5 STRONGLY AGREE	N/A	TOTAL	WEIGHTED AVERAGE
(no label)	0.00%<br>0	3.23%<br>1	0.00%<br>0	38.71%<br>12	58.06%<br>18	0.00%<br>0	31	4.52

Q11 Using Microsoft Teams as a meeting and information platform worked well

Answered: 31 Skipped: 6

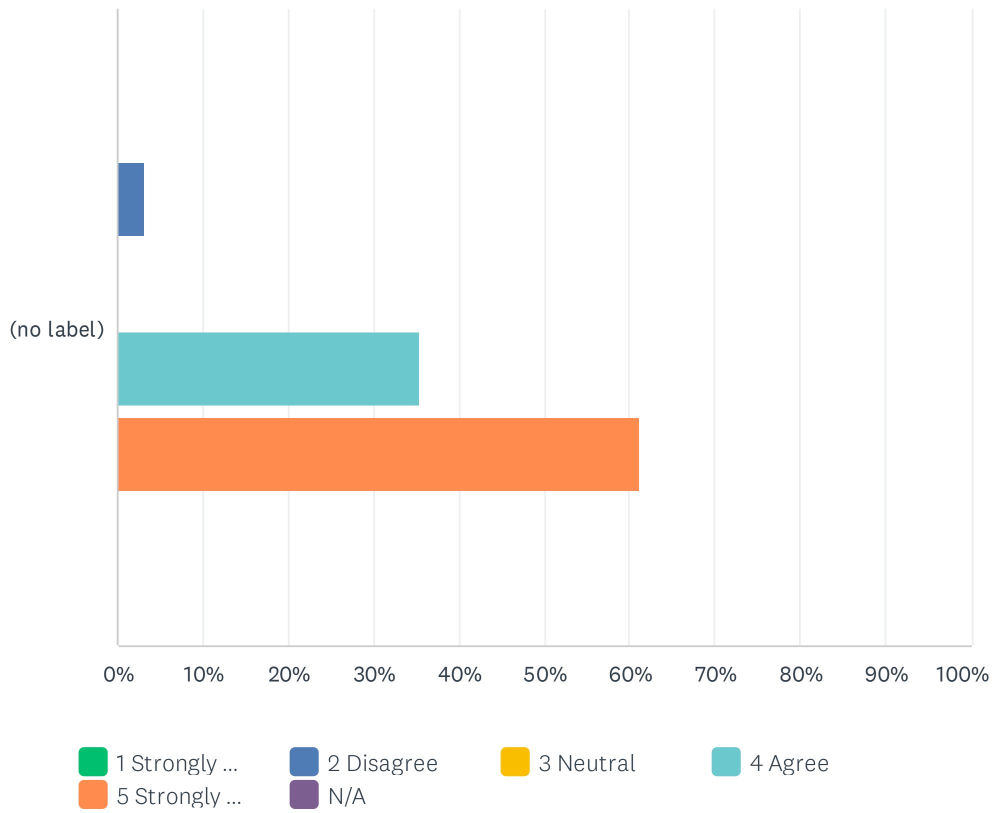

	1 STRONGLY DISAGREE	2 DISAGREE	3 NEUTRAL	4 AGREE	5 STRONGLY AGREE	N/A	TOTAL	WEIGHTED AVERAGE
(no label)	0.00%<br>0	3.23%<br>1	0.00%<br>0	35.48%<br>11	61.29%<br>19	0.00%<br>0	31	4.55

Q12 The logging of my progress and submitting my completed tasks to OSLER was clear and easy to use.

Answered: 31   Skipped: 6

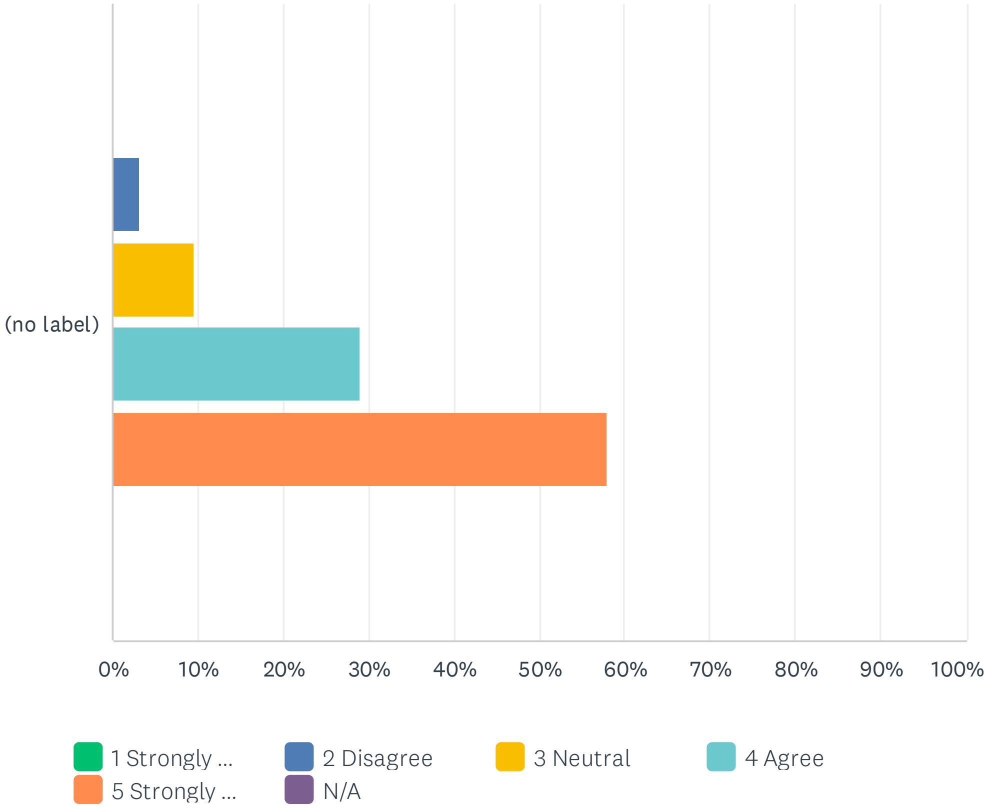

	1 STRONGLY DISAGREE	2 DISAGREE	3 NEUTRAL	4 AGREE	5 STRONGLY AGREE	N/A	TOTAL	WEIGHTED AVERAGE
(no label)	0.00%<br>0	3.23%<br>1	9.68%<br>3	29.03%<br>9	58.06%<br>18	0.00%<br>0	31	4.42

Q13 The COVID project helped me explore how COVID-19 has changed in one key area of medical practice

Answered: 31 Skipped: 6

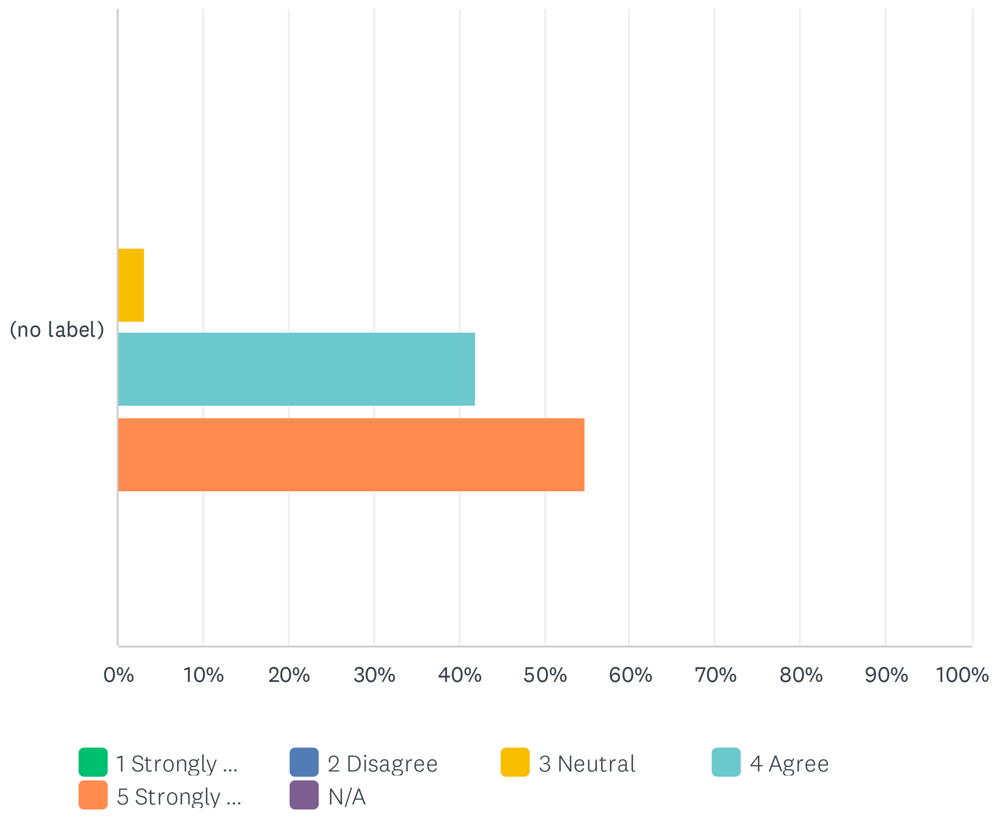

	1 STRONGLY DISAGREE	2 DISAGREE	3 NEUTRAL	4 AGREE	5 STRONGLY AGREE	N/A	TOTAL	WEIGHTED AVERAGE
(no label)	0.00%<br>0	0.00%<br>0	3.23%<br>1	41.94%<br>13	54.84%<br>17	0.00%<br>0	31	4.52

Q14 My project supervisor provided me with timely feedback

Answered: 31 Skipped: 6

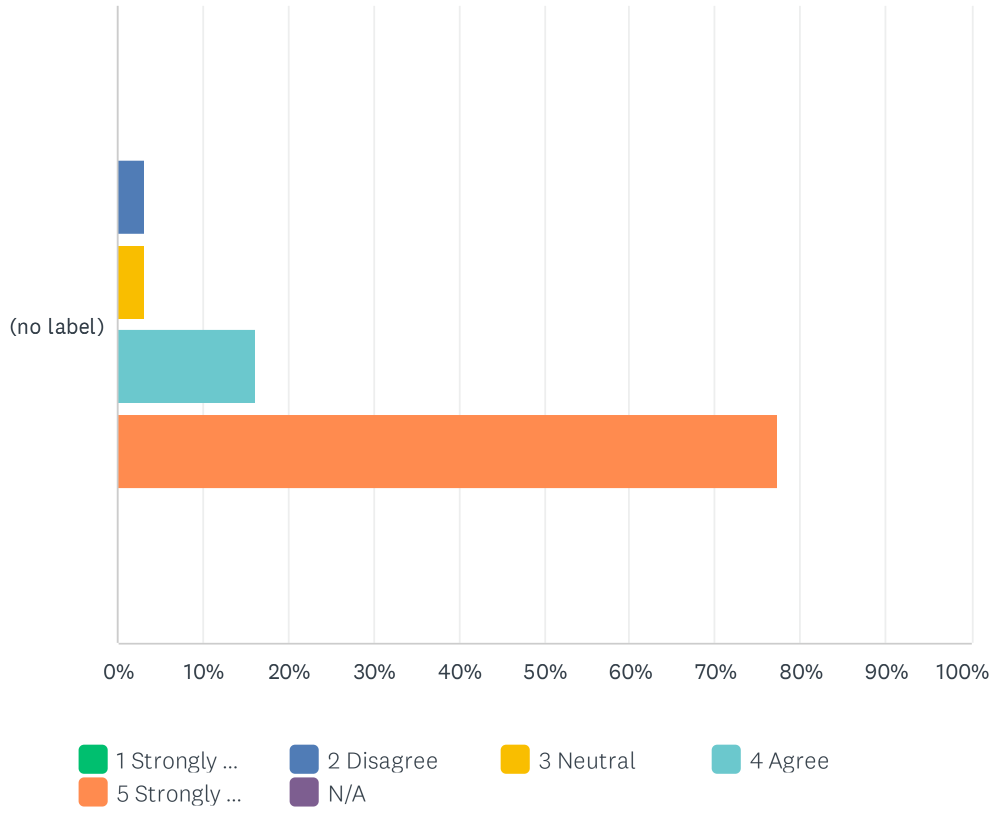

	1 STRONGLY DISAGREE	2 DISAGREE	3 NEUTRAL	4 AGREE	5 STRONGLY AGREE	N/A	TOTAL	WEIGHTED AVERAGE
(no label)	0.00%<br>0	3.23%<br>1	3.23%<br>1	16.13%<br>5	77.42%<br>24	0.00%<br>0	31	4.68

Q15 The 8 options for COVID projects met my needs

Answered: 31 Skipped: 6

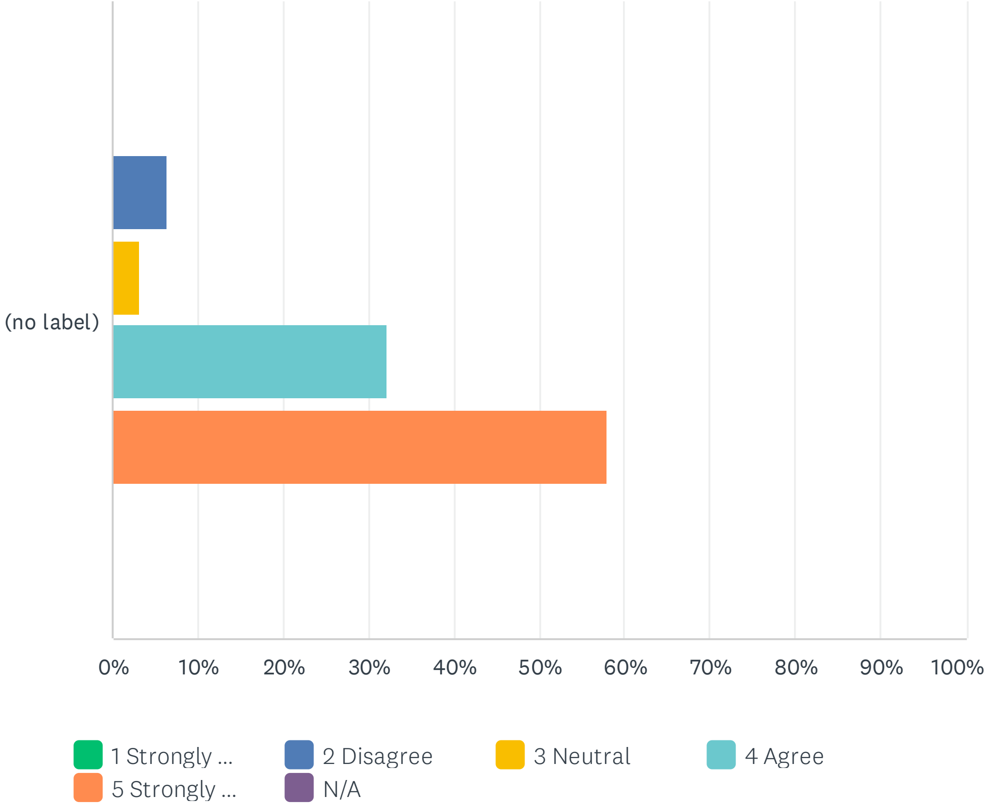

	1 STRONGLY DISAGREE	2 DISAGREE	3 NEUTRAL	4 AGREE	5 STRONGLY AGREE	N/A	TOTAL	WEIGHTED AVERAGE
(no label)	0.00%<br>0	6.45%<br>2	3.23%<br>1	32.26%<br>10	58.06%<br>18	0.00%<br>0	31	4.42

Q16 I enjoyed undertaking the COVID e-lective as an option for a disrupted placement

Answered: 31 Skipped: 6

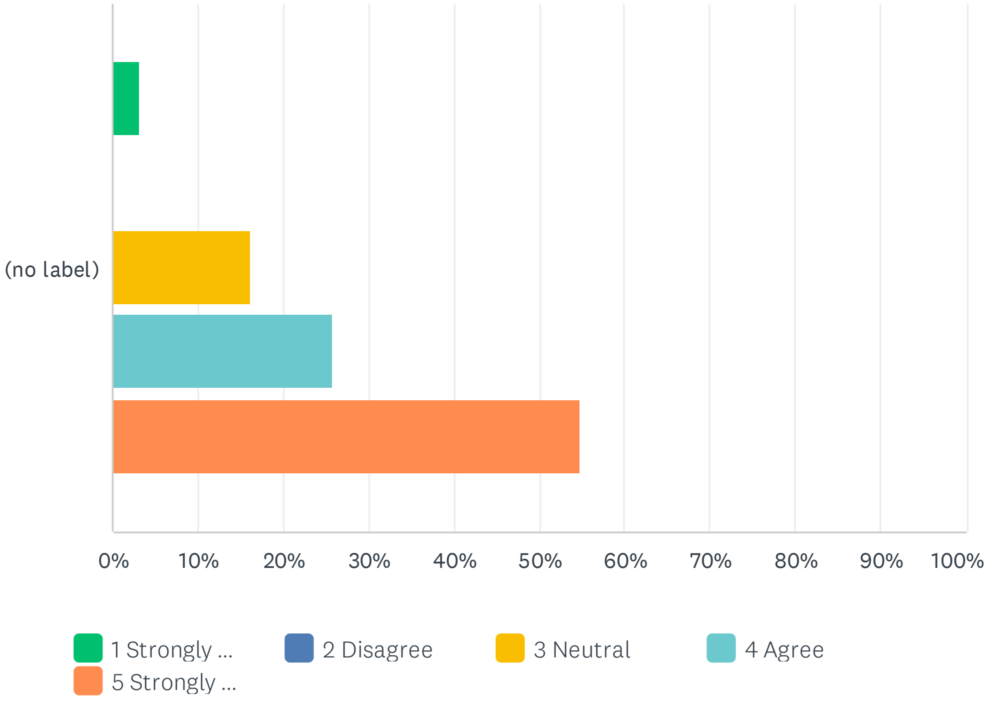

	1 STRONGLY DISAGREE	2 DISAGREE	3 NEUTRAL	4 AGREE	5 STRONGLY AGREE	TOTAL	WEIGHTED AVERAGE
(no label)	3.23%<br>1	0.00%<br>0	16.13%<br>5	25.81%<br>8	54.84%<br>17	31	4.29

Q17 Overall the COVID e-lective was well coordinated

Answered: 31 Skipped: 6

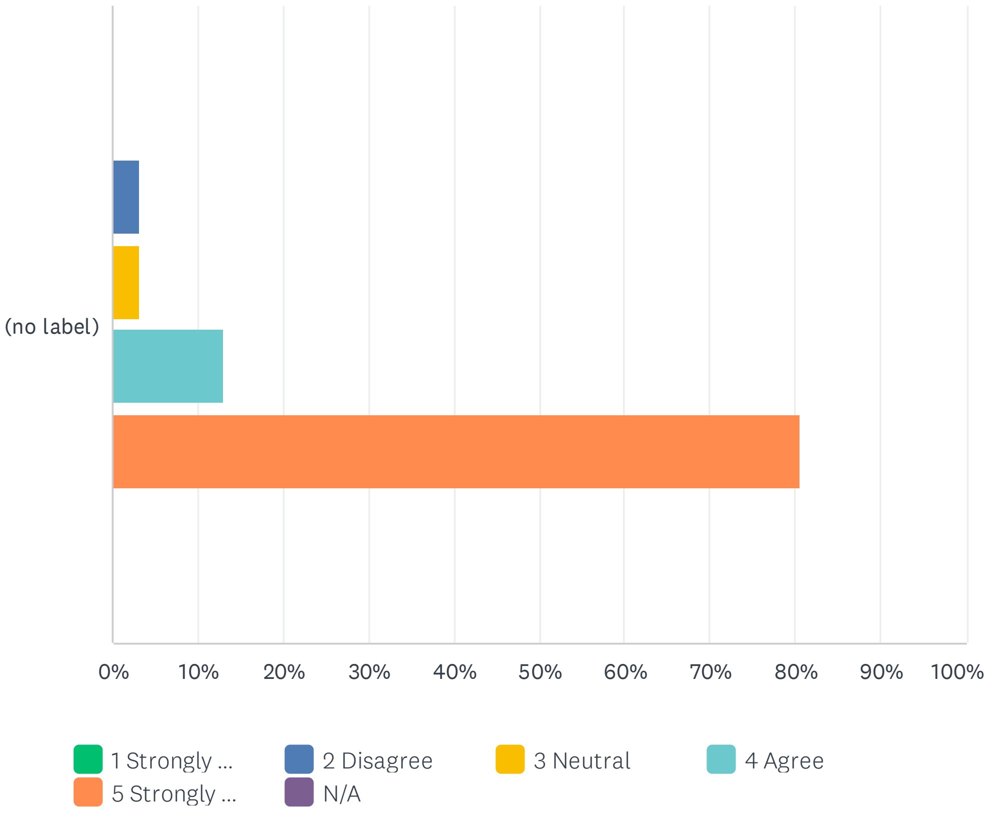

	1 STRONGLY DISAGREE	2 DISAGREE	3 NEUTRAL	4 AGREE	5 STRONGLY AGREE	N/A	TOTAL	WEIGHTED AVERAGE
(no label)	0.00%<br>0	3.23%<br>1	3.23%<br>1	12.90%<br>4	80.65%<br>25	0.00%<br>0	31	4.71

Q18 Overall the COVID e-lective worked well.

Answered: 31 Skipped: 6

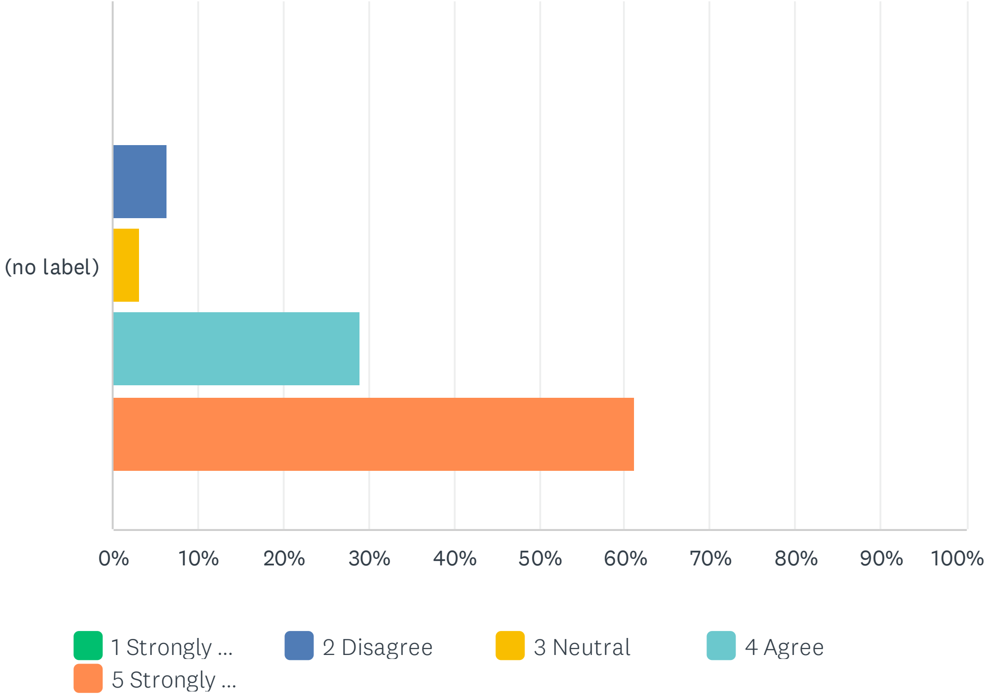

	1 STRONGLY DISAGREE	2 DISAGREE	3 NEUTRAL	4 AGREE	5 STRONGLY AGREE	TOTAL	WEIGHTED AVERAGE
(no label)	0.00%<br>0	6.45%<br>2	3.23%<br>1	29.03%<br>9	61.29%<br>19	31	4.45

Q19 What two things did you enjoy most about the COVID e-lective?

Answered: 24   Skipped: 13

Q20 What two things would you like to see improved?

Answered: 21 Skipped: 16
